# Undergirding CNAs in LTC: The Experience of Collaborative LN-CNA Caregiving Pairs

**DOI:** 10.1093/geroni/igab046.937

**Published:** 2021-12-17

**Authors:** Cynthia Beynon, Katherine Supiano, Elena Siegel, Linda Edelman, Connie Madden, Sara Hart

**Affiliations:** 1 Weber State University, Ogden, Utah, United States; 2 University of Utah, Salt Lke City, Utah, United States; 3 University of California, Davis, Sacramento, California, United States; 4 University of Utah College of Nursing, Salt Lake City, Utah, United States; 5 University of Utah, Salt Lake city, Utah, United States; 6 University of Utah, SALT LAKE CITY, Utah, United States

## Abstract

This research explores support provided by licensed nurses (LNs) to certified nurse aide (CNA) coworkers in the nursing home (NH). Using purposive sampling, we interviewed 12 LN and 12 CNA participants individually and as part of an LN/CNA caregiving pair. Semi-structured interviews were recorded, transcribed verbatim, and coded for meaning using NVivo software. LN and CNA participants described anticipated and unanticipated holistic support for CNAs. We applied the term undergirding to this phenomenon , and we present descriptions and examples of undergirding in nine categories: listen and respond, show respect, help with resident care and answer call lights, protect the CNA, support physical needs, and provide emotional support. Undergirding promotes work success for the CNA and the LN, as the LN job includes oversight of CNA responsibilities. Most notably, participants report undergirding facilitates high-quality resident care. These findings may be helpful for educators and administrators, but perhaps are most important for policymakers. CNAs need additional support to decrease turnover, improve retention, and elevate NH residents' quality of care. The study design identified and explored optimal collaboration as it is possible in the current NH setting. It does not represent all LN/CNA caregiving pairs.

